# The Healthy Heart-Mind trial: melatonin for prevention of delirium following cardiac surgery: study protocol for a randomized controlled trial

**DOI:** 10.1186/s13063-016-1163-1

**Published:** 2016-01-28

**Authors:** Andrew H. Ford, Leon Flicker, Jurgen Passage, Bradley Wibrow, Matthew Anstey, Mark Edwards, Osvaldo P. Almeida

**Affiliations:** WA Centre for Health and Ageing, Centre for Medical Research, Harry Perkins Institute of Medical Research and School of Psychiatry and Clinical Neurosciences, University of Western Australia, Australia, 35 Stirling Highway, Crawley, WA 6009 Australia; WA Centre for Health and Ageing, Centre for Medical Research, Harry Perkins Institute of Medical Research and School of Medicine and Pharmacology, University of Western Australia, Perth, WA Australia; Department of Cardiothoracic Surgery, Sir Charles Gairdner Hospital, Perth, WA Australia; Intensive Care Unit, Sir Charles Gairdner Hospital, Perth, WA Australia; Department of Cardiothoracic Surgery, Fiona Stanley Hospital, Perth, WA Australia

**Keywords:** Melatonin, Delirium, Prevention, Cardiothoracic, Surgery

## Abstract

**Background:**

Delirium is a common occurrence in patients undergoing major cardiac surgery and is associated with a number of adverse consequences for the individual, their family and the health system. Current approaches to the prevention of delirium include identifying those at risk together with various non-pharmacological and pharmacological strategies, although the efficacy of these is often modest. Emerging evidence suggests that melatonin may be biologically implicated in the development of delirium and that melatonin supplementation may be beneficial in reducing the incidence of delirium in medical and surgical patients. We designed this trial to determine whether melatonin reduces the incidence of delirium following cardiac surgery compared with placebo.

**Methods/Design:**

The Healthy Heart-Mind trial is a randomized, double-blind, placebo-controlled clinical trial of 3 mg melatonin or matching placebo administered on seven consecutive days for the prevention of delirium following cardiac surgery. We will recruit 210 adult participants, aged 50 and older, undergoing elective or semi-elective cardiac surgery with the primary outcome of interest for this study being the difference in the incidence of delirium between the groups within 7 days of surgery. Secondary outcomes of interest include the difference between groups in the severity and duration of delirious episodes, hospital length of stay and referrals to mental health services during admission. In addition, we will assess differences in depressive and anxiety symptoms, as well as cognitive performance, at discharge and 3 months after surgery.

**Discussion:**

The results of this trial will clarify whether melatonin reduces the incidence of delirium following cardiac surgery.

**Trial registration:**

The trial is registered with the Australian Clinical Trials Registry, trial number ACTRN12615000819527 (10 August 2015).

**Electronic supplementary material:**

The online version of this article (doi:10.1186/s13063-016-1163-1) contains supplementary material, which is available to authorized users.

## Background

Delirium is a syndrome characterized by cognitive impairment and attention deficits that usually develop over a short period of time and fluctuate in intensity and presentation. Several behavioral and psychological symptoms can accompany delirium, such as agitation, psychosis, disturbed sleep and mood changes, all of which can cause distress to patients, their families and medical staff [1]. Delirium has a prevalence of 15 % among hospitalized older adults [2], although it goes unrecognized in 32–66 % of them [3]. Delirium is particularly common after surgery and is estimated to occur in up to 42 % of patients undergoing major cardiac procedures [4–7].

Delirium is associated with poor clinical outcomes, including higher risk of dementia and admission to residential care, as well as increased mortality [8]. Delirious patients experience more postoperative complications [4], higher readmission rates, poorer functional outcomes [9], and increased length of stay [10]. The annual cost of delirium to the health care system in the United States is estimated to fall between US$38 billion and US$152 billion [11]. Delirium may persist for several weeks in up to a third of patients, leading to further increased morbidity and mortality [12].

Postoperative cognitive decline following cardiac surgery is estimated to occur in 24 to 79 % of patients and persistence of symptoms is common [13]. Newman and colleagues followed 261 patients for 5 years after coronary artery bypass grafting (CABG) surgery [14]. They found that 53 % of participants exhibited cognitive decline at discharge, and although this proportion decreased at the 6-month follow-up (24 %), the prevalence rose again after 5 years to 42 %. These findings are consistent with the possibility that postoperative cognitive dysfunction (of which delirium is an important contributing factor) worsens long-term cognitive outcomes [15].

Various factors increase the risk of delirium, including age, pre-existing cognitive impairment, disruption of the circadian rhythm, dehydration, malnutrition, and certain medications [16]. Major cardiac surgery increases the risk of delirium, with critical contributing factors including the type of surgery, use of hemofiltration, medications administered, length of surgery, time on bypass, high transfusion requirement, postoperative hypertension, patient’s age and whether the surgery was elective [7]. Some of these risk factors are amenable to change, whilst others (e.g., age) are not.

Current approaches to the prevention of delirium in hospitalized patients consist of both non-pharmacological and pharmacological approaches, of which the former are arguably more effective [17]. Non-pharmacological approaches involve addressing multiple risk factors in a systematic manner together with education and environmental manipulation, although only relatively modest reductions in delirium incidence are usually attainable [18]. Pharmacological approaches may include treatment with antipsychotic drugs, cholinesterase inhibitors, hypnotics, anti-inflammatory drugs and gabapentin [19], but supportive evidence of efficacy is not compelling. In addition, all of these agents carry a relatively high risk of adverse effects.

### Evidence linking melatonin and delirium

Melatonin is a serotonin-derived hormone secreted by the pineal gland. It plays a pivotal role in the regulation of the sleep-wake cycle [20], and has numerous other biological and physiological functions. One of the typical features of delirium is the disruption of the sleep-wake cycle and circadian rhythm [1]. This raises the possibility that melatonin-related pathways may mediate the expression of delirium [21]. In addition, older people are more prone to sleep disorders arising from degeneration of suprachiasmatic nuclei and resulting lower melatonin levels [22]. Serum melatonin levels decrease post surgery [23] and after the administration of certain opioids [24], possibly contributing to the disrupted sleep-wake cycle and increased risk of delirium that these patients experience. Plasma melatonin levels have been shown to be lower in delirious patients following major surgery [25] and urine concentrations of melatonin metabolites are altered in people who are delirious [26]. It is also possible that melatonin demand is higher after surgery, thereby leading to a relative depletion and greater risk of delirium [27].

Melatonin secretion is stimulated by low exposure to light and peak production corresponds to the main sleep period in humans. Melatonin is metabolized in the liver and excreted by the kidneys and has a short half-life (40–50 minutes) [28]. The safety profile of melatonin in clinical trials is comparable to placebo [29].

### Existing preliminary data suggest that melatonin might prevent and treat delirium

**Fig. 1:**
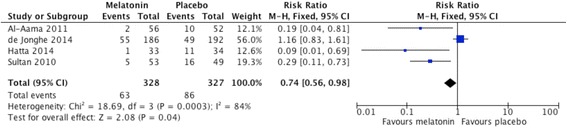
Meta-analysis of available clinical trials. Forest plot showing the published clinical trials of melatonin for the prevention of delirium (primary outcome of incident delirium). The meta-analysis shows a slight benefit for melatonin over placebo although small sample size and study heterogeneity prevents firm conclusions from being drawn

Four clinical trials of melatonin or melatonin agonists for the prevention of delirium have been performed to date (summarized in Fig. 1), although none of these have been conducted in cardiac surgery patients. The earliest of these enrolled 300 patients undergoing hip arthroplasty under spinal anesthesia [30]. Participants were randomly allocated to one of four groups: control, melatonin 5 mg, clonidine and, lastly, a group given midazolam. The melatonin group received one dose in the evening prior to surgery and 90 minutes pre-operatively. The author reported a significant decrease in the prevalence of postoperative delirium in the melatonin group compared with placebo (9.4 % versus 32.7 %, *p* = 0.003; numbers needed to treat (NNT) = 5). Moreover, treatment with melatonin was associated with remission of delirium in 58.1 % of postoperative patients.

In a trial of acutely ill medical in-patients aged 65 and older, Al-Aama and colleagues randomly allocated 72 patients to melatonin and 73 to placebo [31]. Patients consumed 0.5 mg of melatonin or placebo every night for 14 days, or until discharge. Delirium was diagnosed according to the Confusion Assessment Method (CAM) [32]. Melatonin use was associated with a lower risk of delirium (12 % versus 31 %, *p* = 0.014; NNT = 6), with an odds ratio (OR) adjusted for dementia and comorbidities of 0.19 (95 % CI 0.06 to 0.62). These differences remained significant when patients with prevalent delirium on admission were excluded (19.2 % in the placebo and 3.6 % in the melatonin group, *p* < 0.02).

More recently de Jonghe and colleagues completed a multicenter, placebo-controlled clinical trial in 378 patients undergoing hip surgery [33]. Participants received 3 mg of melatonin for 5 days starting within 24 hours of admission. The authors found no difference in the incidence of delirium between the melatonin (55/186, 29.6 %) and placebo (49/192, 25.5 %) groups (difference 4.1 %, 95 % CI −0.05 to 13.1), but reported that a smaller proportion of participants in the melatonin group experienced a long-lasting episode of delirium (>2 days) (25.5 % versus 46.9 %, *p* = 0.02). Lastly, Hatta and colleagues conducted a small trial of the melatonin agonist, ramelteon (8 mg per day for 7 days) in 67 acutely unwell medical patients [34]. They reported a significant decrease in the incidence of delirium in those taking ramelteon as compared to placebo (3 % versus 32 %, *p* = 0.003; NNT = 4). The forest plot presented in Fig. 1 summarizes the efficacy data of these trials.

The results of these trials suggest a potential role for melatonin in preventing and treating delirium in older adults, although conflicting results, methodological issues (e.g., absence of a placebo-control group) and differing patient populations prevent firm conclusions from being drawn and generalized.

### Rationale for the proposed study

Delirium is frequent after cardiac surgery and is associated with many adverse consequences. Emerging evidence suggests that melatonin may be biologically implicated in the development of delirium and that melatonin use may be beneficial in reducing the incidence of delirium in medical and surgical patients, but these results require replication in differing at-risk populations.

### Objectives

The primary outcome of interest for this study is the difference in the incidence of delirium (as determined by the CAM and *Diagnostic and Statistical Manual of Mental Disorders, version 5* (DSM-5) criteria) between the placebo and melatonin groups within 7 days following cardiac surgery. Secondary outcomes of interest include the difference between groups in the severity and duration of delirious episodes, hospital length of stay and referrals to mental health services during the admission. In addition, we will assess for differences in mood and anxiety symptoms and cognitive performance at discharge and at 3 months after surgery.

### Trial design

The Healthy Heart-Mind trial is a randomized, double-blind, placebo-controlled clinical trial of 3 mg melatonin or matching placebo administered on seven consecutive days for the prevention of delirium following cardiac surgery (two arms, 1:1 allocation, parallel design). This study follows SPIRIT guidelines, and the relevant checklist is available as an additional electronic file (Additional file 1). 

## Methods

### Participants and setting

We will recruit 210 participants undergoing elective or semi-elective cardiac surgery in Perth, Western Australia. All outpatients undergoing elective cardiac surgery attend the pre-assessment clinics before admission (usually a week prior). Potential participants will be approached during this clinical review and offered an opportunity to participate in the study. In addition, we will approach patients admitted to cardiology and general hospital wards awaiting surgery on a semi-elective basis; e.g., stable patients admitted with a myocardial infarction or unstable angina deemed suitable for surgical intervention.

### Eligibility criteria

Participants will be:

1. Aged 50 years and older
2. Undergoing elective or semi-elective cardiac surgery (on or off cardiopulmonary bypass) for coronary artery bypass grafting (CABG) and/or valvular surgery

We will exclude participants who:

1. Decline or are unable to give informed consent
2. Are undergoing emergency surgery
3. Are not fluent in written or spoken English (for the purposes of being able to complete the study assessments and questionnaires)
4. Have a contraindication to taking melatonin, such as a prior allergic reaction
5. Are currently using melatonin
6. Have a diagnosis of dementia or score ≤19 on the Telephone Interview for Cognitive Status-modified (TICS-m) [35]
7. Have a score ≥15 on the Alcohol Use Disorders Identification Test (AUDIT) [36]

### Interventions

Eligible participants will be randomly allocated to treatment with 3 mg melatonin or matching placebo for seven consecutive nights, beginning 2 nights prior to the surgery. The contents of each capsule will be administered via a nasogastric tube for those who are unable to swallow.

### Outcomes

We will use a set of well-validated instruments and scales to assess for the presence of delirium and measure its severity, as well as to assess other outcomes of interest for this study.

#### Establishing the presence of delirium

The CAM will be used to establish the presence of delirium. The first assessment will take place in the pre-assessment clinic or hospital ward prior to the surgery (usually a week before but this can vary) and then daily, beginning 1 day post surgery and continuing for up to 7 days or until discharged (whichever comes first). The CAM was developed in 1988–1990 and is a widely used and well-validated screening tool for delirium, with sensitivity of 94 % (95 % CI 91–97 %) and specificity of 89 % (95 % CI 85–94 %) [37]. A trained research nurse will rate the scale on the basis of a standardized interview with the patient that includes a brief cognitive assessment. Case notes and nursing staff reports will be reviewed as part of the assessment. The diagnosis of delirium will be confirmed in consultation with an experienced psychogeriatrician (AHF or OPA). The CAM rates nine areas (acuity, attention, thought organization, level of consciousness, orientation, memory, perception, psychomotor tempo and sleep), but delirium is only diagnosed when there is evidence of acute onset, inattention, fluctuating mental state and either disorganized thinking or disturbed consciousness. The CAM has been successfully adapted for use in the intensive care setting [38]. The rating of the CAM takes about 10 minutes to complete.

#### Establishing the severity of delirium

The Memorial Delirium Assessment Scale (MDAS) [39] will be used to assess the severity of delirium and will be administered daily if delirium is present. The MDAS is a 4-point, 10-item (range 0–30) clinician-rated scale designed to quantify the severity of delirium in a medically ill patient population. It takes approximately 10 minutes to complete.

#### Measuring morbidity and lifestyle

1. Alcohol abuse or dependency: the AUDIT is a 10-item questionnaire designed to screen for excessive drinking. It takes 2 minutes to complete, with scores of 15 or greater indicating harmful alcohol use or dependency [36]
2. Medical morbidities, medications and lifestyle: we will collect information about demographics, lifestyle (smoking, alcohol use, body mass index), common medical conditions diagnosed by a doctor, and medications used (including use of illicit substances). This questionnaire takes approximately 5–10 minutes to complete
3. Details of surgery: we will record the type of surgery and total length of surgical procedure including duration on bypass (if applicable)
4. Medications: we will record participants’ medications on admission and discharge. We will also record the use of all psychiatric medications during hospitalization
5. Referral to mental health or geriatric services during admission will be recorded
6. Length of stay (LOS): we will record total hospital stay duration and intensive care unit (ICU) length of stay in hours for participants discharged alive from hospital
7. Destination post discharge: we will record destination post discharge for all participants and, for the purposes of analysis, will group them into “discharge to usual place of residence” versus “discharge to other places.” Participants who die following cardiac surgery will form a separate group
8. Cognition: this will be measured with the TICS-m. The TICS-m is a brief, widely used telephone assessment instrument with good reliability and validity to screen for dementia and cognitive impairment in older people [40]. It can be rated in person or by telephone. It consists of 13 items measuring various aspects of cognitive function yielding a total score of 50. Scores ≤27 indicate the presence of clinically significant cognitive impairment. Cognitive function will be measured at baseline, discharge and 3 months after surgery. The TICS-m was chosen to allow us to assess participants’ cognition 3 months after surgery in the comfort of their own homes. We deliberately chose this approach to reduce participant burden and improve study retention. In addition, many Western Australian patients live in country areas so that in-person attendance would be difficult
9. Depressive and anxiety symptoms: we will measure depressive and anxiety symptoms at baseline, discharge and at 3 months with the Hospital Anxiety and Depression Scale (HADS) [41]. This is a self-report scale of 14 items in total that gives sub-scores for both depression and anxiety

### Participant timeline and recruitment

**Fig. 2:**
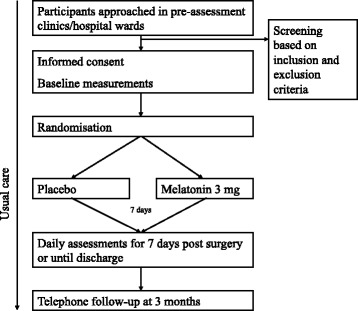
Summary of participant timelines. The figure summarizes the enrollment procedure and time commitment for participants

Table 1 shows the trial assessment schedule and Fig. 2 summarizes the recruitment and the flow of participants in the trial. Participants will be approached in pre-assessment clinics (all patients attend these a week or so prior to the scheduled surgery) and general hospital wards for those already hospitalized and awaiting surgery (i.e., semi-elective cases). Enrolled participants will be seen daily following their surgery with the primary outcome of interest being the difference in the incidence of delirium between the two groups. The final hospital assessment will occur on day 7 following surgery or the day of discharge (whichever comes first). Participants will be followed up one more time at 3 months after surgery to assess for the presence of delirium and to measure cognitive function.

**Table 1:** Summary of trial assessment schedule

	Baseline	Daily	Discharge/7 days post surgery	3 months
Demographics	x			
Medical comorbidities	x			
Medications	x		x	
Surgery details			x	
Referral to mental health and geriatric services			x	
AUDIT	x			
LOS			x	
Adverse events	x		x	
CAM	x	x	x	x
MDAS	x	x (if required)	x (if required)	x (if required)
TICS-m	x		x	x
HADS	x		x	x

*AUDIT* Alcohol Use Disorders Identification Test, *CAM* Confusion Assessment Method, *HADS* Hospital Anxiety Depression Scale, *LOS* length of stay, *MDAS* Memorial Delirium Assessment Scale, *TICS-m* Telephone Interview for Cognitive Status-modified

### Sample size

The primary outcome of the proposed trial is the development of delirium within 7 days of surgery. We based our sample size on the trial of Al-Aama and colleagues [31]. They reported a 15.6 % absolute reduction (19.2 % versus 3.6 %, *p* < 0.02) in the incidence of delirium in their sample. Assuming a 15.6 % absolute risk reduction in our patients with a conservative 25 % incidence of delirium in the control group and an incidence of 9.4 % in the intervention group, we will require 91 participants per group (182 participants in total) to detect a difference of this magnitude with a power of 80 % and a two-sided alpha of 0.05. After allowing for a conservative loss to follow-up of 15 % [31], we calculated that we will need to recruit 210 participants (105 per group). A defined secondary outcome for this trial is cognitive performance 1 week and 3 months after surgery. A study with 100 participants per group at the 3-month follow-up will have 81 % power to declare as significant between group difference of 2 points in the change from baseline score of the TICS-m (1 versus 3 points, SD 5). A change of less than 2 points would be of uncertain clinical significance.

### Allocation, randomization, blinding and compliance

Participants will be randomized to treatment with melatonin or placebo in a ratio of 1:1 according to a list of computer-generated random numbers in permuted blocks ranging from 8 to 12. This strategy decreases the risk of unblinding of participants in one particular block if, for some reason, the group membership of one of its members is disclosed. The list will be generated and maintained by an independent biostatistician.

The pharmacy will dispense melatonin or placebo. Melatonin and placebo will be administered as oral capsules that have the same size, weight, color, texture and taste. The active medication will consist of 3 mg melatonin. These will be manufactured in batches every 12 months with 6-monthly stability testing performed for weight, disintegration, impurities and appearance. The pharmacy will be responsible for dispensing all study medications, which will be included on the patient medication chart as per usual hospital practice. Unblinding will only occur once the final endpoint of interest is collected from the last participant in the trial. Compliance will be encouraged through a telephone call to each participant 2 days prior to his or her scheduled surgery date. Participants will be reminded to take their tablets and will be asked to bring the remainder into hospital for use following their surgery. Participants will be given ten tablets to allow for the possibility of surgery being postponed once they have commenced taking the trial medication. We will record all incidences where participants take extra tablets beyond the scheduled seven. Treatment adherence will be determined by the systematic review of medication charts upon discharge and by pill count. Use of sedative/psychotropic medications outside of the study protocol will also be recorded.

### Data collection and management

Data collection will be paper-based and subsequently entered into a secure, password-protected database. Paper files will be kept in a locked filing cabinet in the principle investigator’s office and stored for a minimum of 5 years following completion of the trial.

### Statistical methods

We will analyze and store the data using reliable statistical software (Stata, StataCorp, College Station, TX, USA). Baseline data will be compared using descriptive statistics, Student *t* tests for normally distributed continuous variables, the Mann-Whitney test for ordinal variables and Pearson’s chi-square statistic for categorical variables. The primary outcome analysis will be intention-to-treat and will use multilevel logistic regression of panel data (xtlogit). This approach to the analysis of the data allows for the inclusion of all available information and for the investigation of interactions between terms (such as intervention and time). A similar approach will be used to analyze other binary outcomes (e.g., use of psychotropic medication). We will use multilevel mixed models to investigate changes in scores of the MDAS and TICS-m (continuous variables). All probability tests will be two-tailed and will be declared as significant when *p* < 0.05.

### Data monitoring and safety

We will monitor adverse effects with a checklist of symptoms commonly associated with experimental medications, such as tremor, headaches, anxiety, insomnia and gastrointestinal symptoms. The participants will be asked about the emergence of any new symptoms since the last assessment and how much they have been bothered by them: not at all, a little, quite a bit and extremely. The checklist will be completed prior to enrollment and at discharge. The investigators will use this to screen for any new symptoms that could be attributed to the study medication. Melatonin is a naturally occurring hormone and the incidence of serious adverse events is expected to be low. An independent, unblinded data monitoring committee, chaired by an independent academic biostatistician, will be established to oversee the safety of participants in the trial. This committee will meet twice per year. Using statistical criteria for acceptable deviations from the null hypothesis, the committee will advise the investigators whether the recruitment can continue or whether the study should be terminated.

### Ethics and dissemination

All participants will be required to provide written informed consent prior to enrollment in the trial. The Human Research Ethics Committee of the Sir Charles Gairdner Hospital, Perth, Western Australia has approved the study protocol. Results of the trial will be widely disseminated in relevant literature and at leading international and national conferences.

## Discussion

Delirium is a major issue for the medical community and is associated with universally poor outcomes for patients, their families and the health care system. Delirium is particularly problematic in surgical populations and may complicate up to 42 % of patients undergoing major cardiac surgery. Identification and management of sufferers is often complex and many individuals are missed. Prevention of this common condition is, therefore, essential. This trial will determine if melatonin is able to reduce the incidence and duration of delirium following cardiac surgery and also reduce the burden of other associated factors such as cognitive decline, hospital length of stay and anxiety and depressive symptoms.

The design of this trial has strengths and weaknesses that merit comment. It has not yet been established what dose of melatonin may be optimal for the prevention or treatment of delirium. The trials conducted to date have utilized a variety of doses ranging from 0.5 to 5 mg [30, 31, 33] and one that used 8 mg of the melatonin agonist ramelteon [34]. The trial by de Jonghe and colleagues showed the 3-mg dose used in the study to be both well-tolerated and safe [33], as did another trial in tracheostomized intensive care patients that additionally showed that 3 mg melatonin successfully increased blood levels 1000-fold [42]. A number of other trials have confirmed the safety and tolerability of a 3-mg dose in populations with varying medical morbidities and age ranges [43–46]. We will exclude participants with cognitive impairment, given the possible difficulties with consent and the ability to follow the trial protocol. We understand, however, that this may reduce the overall incidence of delirium in our sample, as pre-existing cognitive impairment is a strong predictor of delirium.

This trial will be restricted to adults aged 50 and older because the incidence of delirium is higher in later life. We will also exclude participants undergoing emergency surgery because of the ethical requirements for recruitment and informed consent. The study will be limited to participants with sufficient English skills to be able to participate in the assessments and complete the study questionnaires. All of these factors could potentially decrease the generalizability of our findings.

These limitations should be considered in light of a number of strengths of this trial. We will be recruiting from a well-defined population at high risk of developing delirium. The trial has well-defined outcomes and has been adequately powered. We will investigate the impact of melatonin on delirium in the immediate postoperative period and will also be able to study its clinical effects 3 months later.

Melatonin could prove to be a simple, cost-effective and safe intervention for patients at high risk of delirium. The results of this Consolidated Standards of Reporting Trials (CONSORT) compliant trial, if positive, could contribute to change the way people at high risk of delirium are managed in clinical practice.

### Trial status

The Healthy Heart-Mind trial has not yet commenced recruitment.

## Additional file

Additional file 1:
**SPIRIT 2013 Checklist: recommended items to address in a clinical trial protocol and related documents*.** (DOC 114 kb)

## Abbreviations

AUDIT: Alcohol Use Disorders Identification Test
CABG: coronary artery bypass grafting
CAM: Confusion Assessment Method
DSM: *Diagnostic and Statistical Manual of Mental Disorders*
HADS: Hospital Anxiety and Depression Scale
ICU: intensive care unit
LOS: length of stay
MDAS: Memorial Delirium Assessment Scale
SD: standard deviation
TICS-m: Telephone Interview for Cognitive Status-modified
